# Genetic Characterization of Apulian Olive Germplasm as Potential Source in New Breeding Programs

**DOI:** 10.3390/plants8080268

**Published:** 2019-08-05

**Authors:** S. Sion, F. Taranto, C. Montemurro, G. Mangini, S. Camposeo, V. Falco, A. Gallo, G. Mita, O. Saddoud Debbabi, F. Ben Amar, S. Pavan, V. Roseti, M. M. Miazzi

**Affiliations:** 1 Department of Soil, Plant and Food Sciences, University of Bari Aldo Moro, 70121 Bari, Italy; 2 Research Centre for Cereal and Industrial Crops, (CREA-CI), S.S. 71122 Foggia, Italy; 3 Department of Agricultural and Environmental Sciences, University of Bari Aldo Moro, 70121 Bari, Italy; 4 CNR Institute of Sciences of Food Production, Unit of Lecce, 73100 Lecce, Italy; 5 Banque Nationale de Gènes, Charguia 1, Tunis 1080, Tunisia; 6 Institut de l’Olivier, Route de l’aéroport, BP 1087, Sfax 3000, Tunisia

**Keywords:** olive, SSR, genetic diversity, population structure, olive breeding

## Abstract

The olive is a fruit tree species with a century-old history of cultivation in the Mediterranean basin. In Apulia (Southern Italy), the olive is of main social, cultural and economic importance, and represents a hallmark of the rural landscape. However, olive cultivation in this region is threatened by the recent spread of the olive quick decline syndrome (OQDS) disease, thus there is an urgent need to explore biodiversity and search for genetic sources of resistance. Herein, a genetic variation in Apulian olive germplasm was explored, as a first step to identify genotypes with enhanced bio-agronomic traits, including resistance to OQDS. A preselected set of nuclear microsatellite markers allowed the acquisition of genotypic profiles, and to define genetic relationships between Apulian germplasm and widespread cultivars. The analysis highlighted the broad genetic variation in Apulian accessions and the presence of different unique genetic profiles. The results of this study lay a foundation for the organization of new breeding programs for olive genetic improvement.

## 1. Introduction

**Figure 1:**
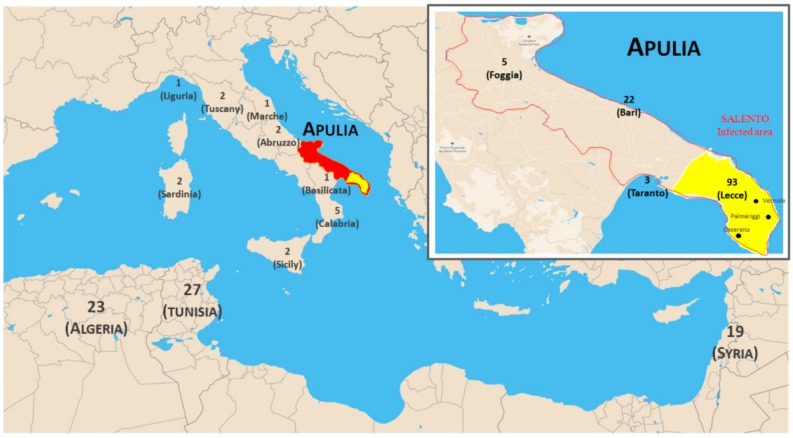
A geographic map of the Mediterranean basin and a zoom of the Apulia region in southern Italy. The numbers indicate the number of accessions sampled in individual Mediterranean countries, in Italian regions and in Apulian provinces. The yellow area highlights the zone of Salento affected by the bacterium X. *fastidiosa* subsp. *pauca*. Three localities (Casarano, Palmariggi and Vernole) are finally reported in which five genotypes asymptomatic to the olive quick decline syndrome (OQDS) disease were collected.

The olive (*Olea europea* L. var. *sativa* Hoffm. e Lk.) is a fruit tree species with remarkable cultural and economic importance [1,2,3]. Its cultivation can be finalized to the production of olive oil and table olives and is of particular relevance for its benefits on human health and rural lifestyles [4,5]. Globally, olive cultivation covers an area of 10.3 million hectares, and is mainly concentrated in Mediterranean countries, which account for more than 95% of the total production. Spain is the major producer of olives in the European Union (59%), followed by Italy, Portugal and Greece (FAOSTAT data 2017, http://www.fao.org/faostat/en/#home). In Italy, olive cultivation is mainly concentrated in the southern part, especially in the region of Apulia [6] (Figure 1). Apulia bases a major part of its agricultural economy on olive cultivation, resulting in 381,550 tons of oil produced in 2018. Approximately 31% of Apulian oil is produced in Salento, the southernmost area of Apulia [7].

The olive is one of the oldest cultivated trees, as it traces back its origins in the Levant region (i.e., eastern Mediterranean) approximately six thousand years ago [8]. Historical information disclosed that, most likely, olives were disseminated in the Mediterranean basin first by the Phoenicians, and then by the ancient Greeks and Romans [9]. Based on fossil/subfossil records and the genetic footprint of plastid DNA polymorphisms, three principal gene pools (namely Q1, Q2 and Q3) were identified for the domesticated olive, corresponding to three main geographical areas: Western (Q1), central Mediterranean (Q2) and eastern Mediterranean (Q3) [10,11].

The olive gene pool refers to thousands of cultivars, wild and feral forms. Italian germplasm includes more than 600 cultivars, even if many homonymies and synonymies are present, due to clonal selection, grafting and migration [12,13].

The study of olive genetic diversity is pivotal to guide the introgression of favourable allelic variants in future breeding programs, to assure food safety and protect product authenticity [14,15,16,17]. However, the genetic structure of the Apulian olive population is poorly explored and still debated, and great confusion exists among cultivars and landraces [18]. 

Olive cultivation is affected by climate change due to an increase of evapotranspiration and irrigation requirements and the occurrence of specific emergent pests and diseases [19,20]. Since 2013, a severe disease, named olive quick decline syndrome (OQDS), is affecting the heritage of Apulian olive trees, with an enormous negative impact on the economy and landscape. The disease emerged in a restricted area of the Ionian coast of Salento (South-Western Apulia), however its incidence increased rapidly throughout the olive-grown countryside of the peninsula. The OQDS syndrome is associated with a complex of symptoms that includes rapid twig and branch dieback, associated with the presence of the bacterium *Xylella fastidiosa* subsp. *pauca*. The spread of the disease is favoured by the bacterial vector *Philaenus spumarius* [21,22]. In the region affected by OQDS, olive orchards are 85% composed of the two cultivars Ogliarola salentina and Cellina di Nardò, which have been shown to be particularly susceptible to the disease. On the contrary, the cultivar Leccino, also cultivated in the area, shows an interesting partial resistance to OQDS [23]. The infected Leccino plants show a lower bacterial concentration and a different gene expression profile during the infection, suggesting the occurrence of genetic mechanisms inhibiting bacterial multiplication and diffusion in plants [24]. More recently, the cultivar Fs-17 was reported to show the highest level of resistance to OQDS, associated with approximately half of the bacterial population quantified in infected Leccino plants [25]. Fs-17 is a seedling of Frantoio, an Italian cultivar widespread at the international level and known with many synonyms (https://brevetti.cnr.it/InfoCatalogo.do?nsrif=547&dip=0).

Molecular markers are widely used in crop species, including olives, to dissect genetic diversity, characterize cultivars and identify synonymies and homonymies [26,27,28,29,30]. The random amplified polymorphic DNA (RAPD) and amplified fragmentlLength polymorphism (AFLP) markers were used to dissect genetic relationships among wild forms and cultivars retrieved in several Mediterranean countries [31,32], including Italy [33,34]. The advances in next generation sequencing (NGS) approaches nowadays allow the use of other kind of markers, such as single nucleotide polymorphisms (SNPs) [13,35,36,37]. However, the complex structure and the high level of heterozygosity of the olive genome makes simple sequence repeat (SSR) markers a primary choice to investigate genetic variation in olive germplasm collections due to their versatility and informativeness related to high repeatability, codominant nature and specificity [26,27,38]. Therefore, SSRs have been repeatedly used in studies addressing the genetic characterization and traceability of olive germplasm [39,40,41,42,43]. 

In this study, the genetic variability of Apulian germplasm is explored, especially the one cultivated in Salento, as a first step for the identification of adaptation to environmental conditions and displaying enhanced bio-agronomic traits, including tolerance to diseases and pests. Synonymies and genetic relationships with Italian and international olive germplasm were established. The unknown accessions showing no OQDS symptoms and thus possibly tolerant to the disease were identified and characterized at the genetic level.

## 2. Results

### 2.1. Allelic Variation of SSR Markers 

The genetic variation among 218 olive cultivars was estimated using nine SSR markers. A total number (Na) of 189 alleles, ranging from 12 to 32 for the DCA05 and DCA18 loci, respectively, corresponding to an average of 21 alleles per locus, were detected (Table 1). Moreover, an effective number (Ne) of 69.78 alleles was obtained, ranging from 3.49 for EMOL to 13.63 for DCA09. Wide genetic variation was observed, as indicated by the high values of observed (Ho) and expected (He) heterozygosity. Ho ranged from 0.32 to 0.88 for the loci EMOL and DCA03, respectively, and was associated with an average value of 0.68. He ranged from 0.71 to 0.93 for the loci EMOL and DCA09 respectively, and was associated with an average value of 0.85. The mean observed heterozygosity was lower than the mean expected heterozygosity, determining a positive fixation index (F) for all the loci (mean F = 0.20), except for DCA03 (F = −0.02) (Table 1).

The calculation of the polymorphic information content (PIC) index, ranging from 0.69 to 0.92 for EMO90 and EMOL, respectively, highlighted good discriminating power for all the markers. The PIC values were high even when considering germplasm for each of the five Mediterranean countries considered in this study (Supplementary Table S1), although Tunisian, Algerian and Syrian populations showed slightly lower PIC values of 0.60, 0.64 and 0.57, respectively. The average F values were positive for all the populations except for the Syrian one that showed an average F value of −0.04 (Supplementary Table S1).

### 2.2. Genetic Characterization of Olive Germplasm

The genetic population structure was assessed through three different approaches, in order to validate results and define robust relationships among olive cultivars. 

**Figure 2:**
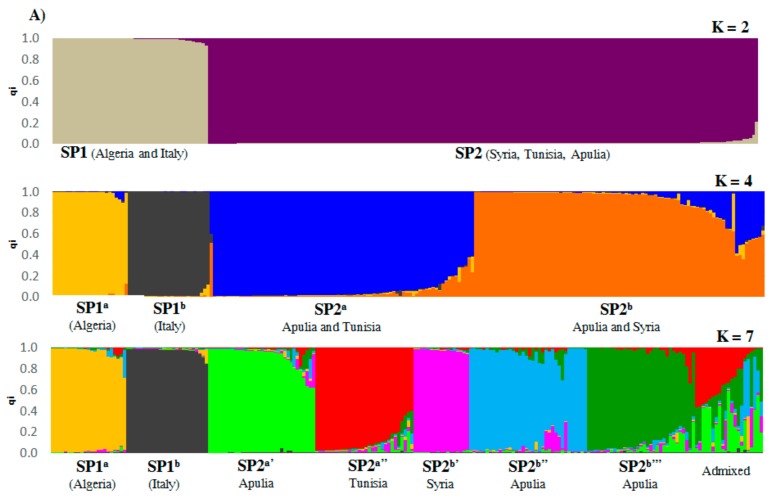

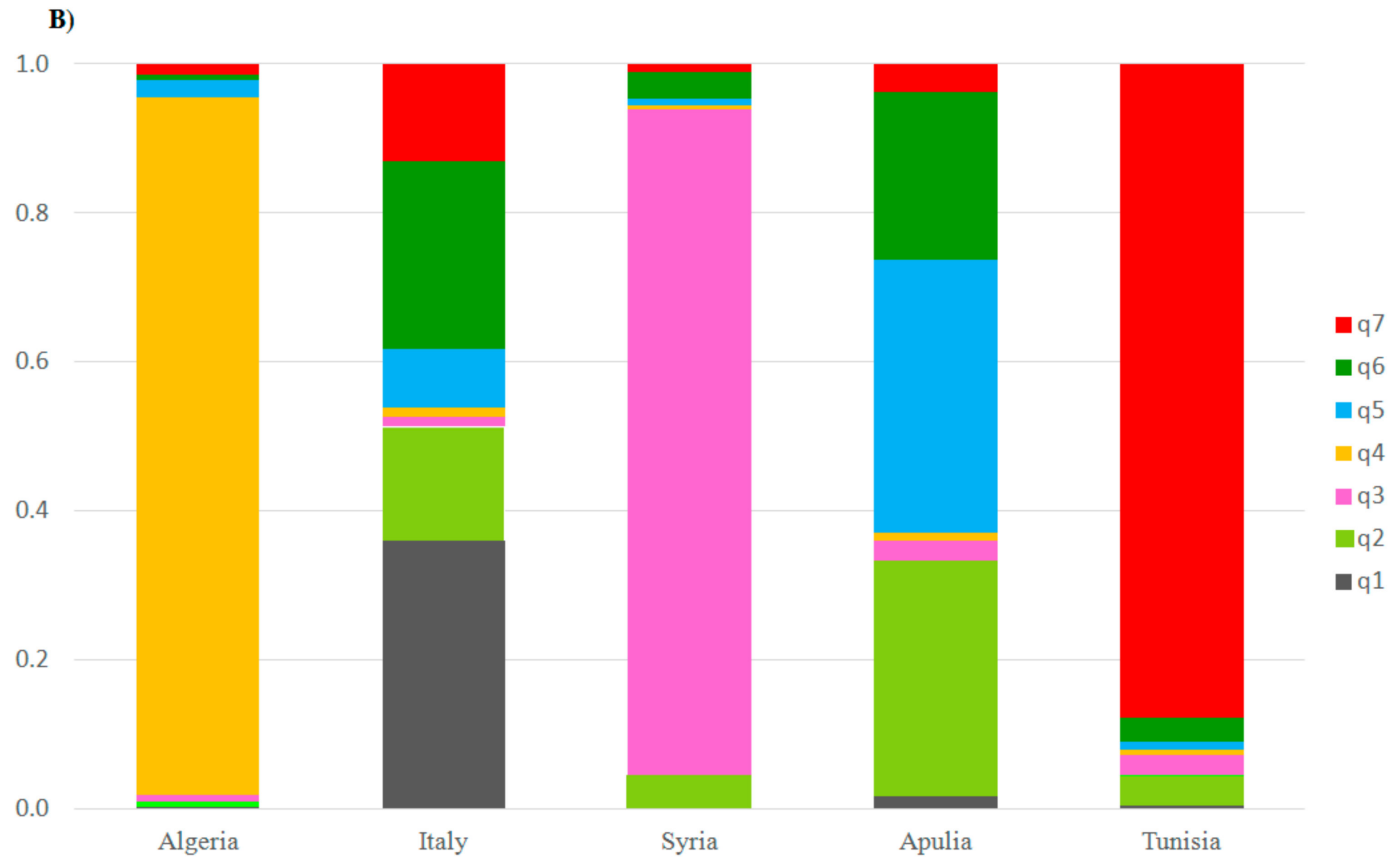
(**A**) The genetic structure of 218 olive accessions identified by the STRUCTURE algorithm at K = 2, K = 4 and K = 7; (**B**) The stacked bar plots showing, for olive populations originating from different geographical areas, the estimated membership coefficient (qi) relative to the subpopulations identified by STRUCTURE for K = 7.

The application of the Bayesian clustering method implemented by the software STRUCTURE indicated that a number of subpopulations (K) of 7 best fits the data, immediately followed by K = 2 and K = 4 (Figure 2A; Supplementary Figure S1a,b, Supplementary Table S2). At K = 2, the olive collection is clearly divided into two sub-populations (hereinafter referred to as SPs): SP1 and SP2. SP1 includes Algerian cultivars and the most common Italian cultivars, while SP2 groups includes Apulian, Syrian and Tunisian germplasm. At K = 4, both SP1 and SP2 are split into two sub-groups. The sub-groups SP1^a^ and SP1^b^ include Algerian and Italian cultivars, respectively. The subgroup SP2^a^ encompasses part of the Apulian germplasm and Tunisian cultivars, whereas the subgroup SP2^b^ includes the remaining part of the Apulian germplasm and Syrian cultivars. At K = 7, the SP2 subgroups were further split. SP2^a’^ includes widespread Italian cultivars such as Taggiasca, Ogliarola Salentina and Cima di Mola, together with some Apulian accessions. SP2^a’’^ clusters three Italian cultivars (Semidana, Martellini and Ascolana tenera) and all Tunisian cultivars, except for two falling in the admixed group (Hawaria and Zarrazi). The last three groups are (SP2^b’^) composed by Syrian cultivars (except for Dan and Kayssy, included in SP2^a^’ and in the admixed group, respectively), and two (SP^2b’’^ and SP2^b’’’^) including Apulian genotypes. Interestingly, one of the five unknown genotypes asymptomatic to the OQDS falls into SP2^b’’’^, together with two accessions of the resistant cultivar Fs-17, while the remaining four unknown asymptomatic genotypes are grouped in SP2^a^’, together with Leccino. Considering the mean qi, which is the estimated membership coefficient for each of five populations *a priori* defined based on geographical origin, a different genetic stratification (Figure 2B) was observed. In particular, the groups of Algerian, Tunisian and Syrian accessions show a proportion of qi greater than 0.85, while the proportions of qi were admixed in Italian (national) and Salento groups. 

**Figure 3:**
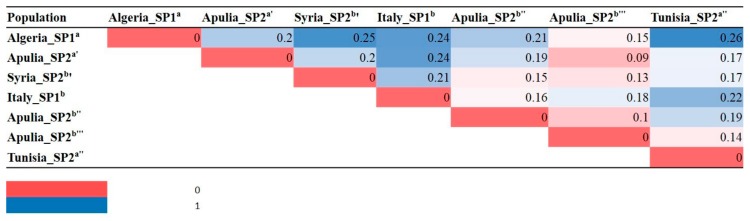
Genetic differentiation (F_ST_) between subpopulations detected by STRUCTURE at K = 7.

The calculation of the pairwise F_ST_ distances among subpopulations identified by STRUCTURE at K = 7 highlighted great genetic differentiation between SP1^b^ and SP2^b’^ (F_ST_ = 0.25), and between SP1^b^ and SP2^a’’’^ (F_ST_ = 0.26) (Figure 3). Conversely, the lowest F_ST_ distances were found between SP2^b’’’^ and SP1^a^’ (0.09) and between SP2^b’’’^ and SP2^b’’^ (0.10). Overall, SP2^b’’’^ presents the lowest pairwise F_ST_ distance, ranging from 0.09 (with SP2^a’^) to 0.18 (with SP1^b^) (Figure 3).

**Figure 4:**
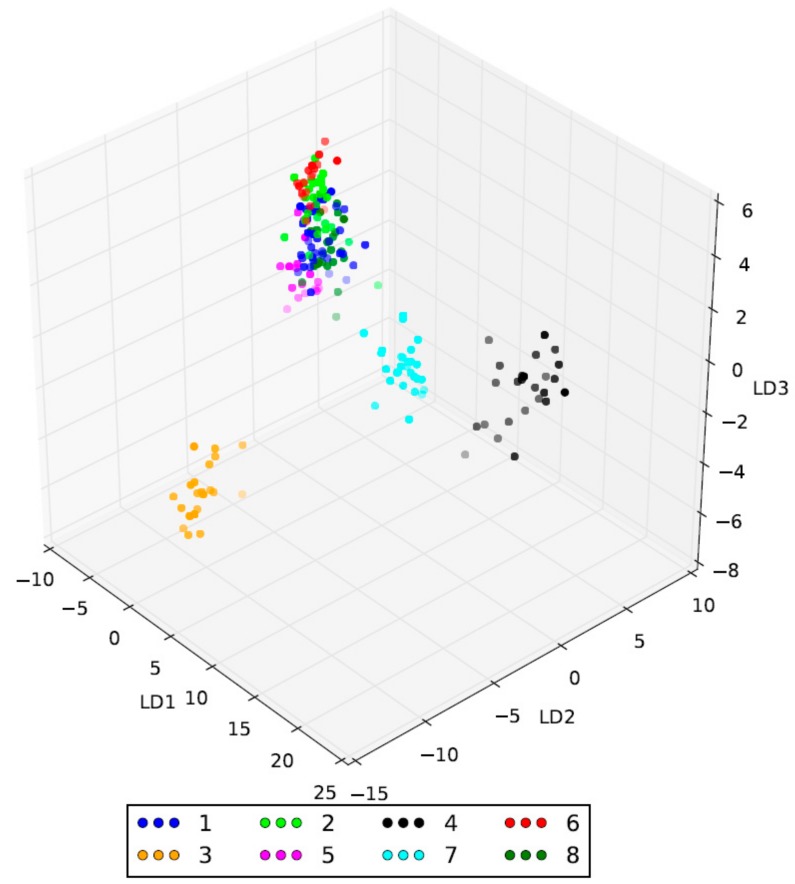
The genetic structure assessed by a discriminant analysis of principal components (DAPC). The 3-D scatter plot is referred to the first three discriminant functions.

To validate clustering obtained by the STRUCTURE analysis, a discriminant analysis of principal components (DAPC) was performed. The application of the Bayesian information criterion (BIC) indicated a number of clusters (K) equal to 8 as the most probable for the data (Supplementary Figure S2a). A bar plot of discriminant analysis eigenvalues associated with the seven linear discriminant functions retained for analysis is shown in Supplementary Figure S2b. For each genotype, the membership coefficients relative to the eight clusters are available in Supplementary Table S2. Clusters 3, 4 and 7 were clearly differentiated using the three main discriminant functions (Figure 4), while clusters 5, 6 and 8 were separated by the fourth, fifth and sixth discriminant functions (Supplementary Figure S3). Cluster 1 includes accessions collected in Salento, some widespread Italian cultivars such as Maiatica, Toscanina, Coratina and Bella di Cerignola, nine Tunisian cultivars and one Syrian cultivar (Kayssy_1). Cluster 2 groups includes genotypes collected in Salento, including four accessions that did not show evident symptoms of the OQDS disease, and some Italian cultivars such as Taggiasca, Ogliarola Salentina, Cima di Bitonto, Cima di Mola and Fs-17. Cluster 3–6 group includes Algerian, Italian, Syrian and Tunisian cultivars, respectively. Lastly, clusters 7 and 8 are formed by the remaining Apulian cultivars. 

**Figure 5:**
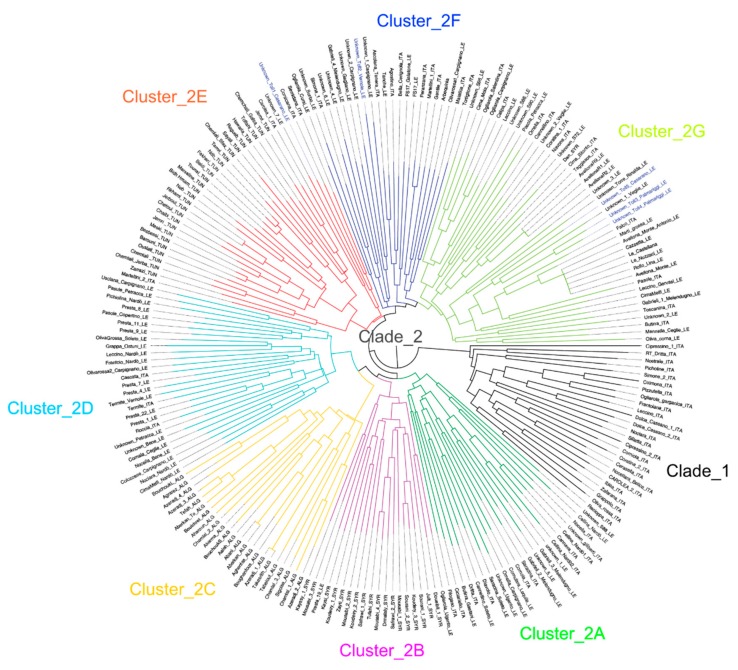
A dendrogram generated by neighbor-joining clustering, illustrating phylogenetic relationships among 218 olive accessions assessed using the SSR markers. The colors of the clades reflect those assigned to STRUCTURE and DAPC clusters. The blue labels indicated five unknown-toll accessions asymptomatic to the OQDS disease.

The dendrogram obtained by neighbor-joining clustering is substantially in agreement with the results of the STRUCTURE and DAPC analyses (Figure 5), and provide further information on genetic relationships among individual olive accessions. The tree root separates two clades, the first (Clade1) mostly including Italian cultivars, and the second (Clade2) clustering genotypes of other origin (Figure 5). The dendrogram partially supports the results of STRUCTURE and DAPC, assigning the Apulian genotypes to three separate clusters, of which two (2A and 2D) close to Algerian and Syrian cultivars, and one (2G) close to Tunisian cultivars. 

### 2.3. Synonymies Discovery

The synonymies were disclosed by hierarchical clustering and the Lynch and Ritland estimator (LRM) analysis (Figure 5, Supplementary Table S3; Supplementary Figure S4). The LRM analysis displayed strong relationships (LRM = 0.50) among the accessions AvellonaR1_LE, AvellonaR2_LE, Avellona_Monte_LE, Rollo_Lina_LE, Le_Nuzzaci_LE, Le_Castellana, Cazzetta_LE and Avellona_Monte_Antonio_LE. Among other relationships, Cima di Mola shares identity with Ogliarola Salentina_ITA, and Ogliarola_garganica_ITA with Frantoiana_ITA (LRM > 0.38). In addition, the two Italian cultivars Semidana_ITA and Corsicana_ITA share a strong similarity with Tunisian cultivars (Figure 5). Only two unknown accessions, Unknown_1_LE and Unknown_S72_LE, were found to have some similarity with the known cultivars Cellina_Nardò2_ITA and Navone_ITA, respectively (LRM > 0.37). The LRM analyses were supported by the cladogram where the other unknown accessions were clearly separated by known cultivars. 

## 3. Discussion

Understanding the basis of biodiversity is the first key step to identify genotypes that best fit the requirements of breeding programs. In Italy, a list of national olive varieties for marketing purposes has been prepared (https://www.politicheagricole.it/flex/cm/pages/ServeBLOB.php/L/IT/IDPagina/10035). However, the lack of a certification system for propagating material has generated confusion among cultivars, with several cases of synonymy and homonymy [10,44]. 

Herein, this study examined the genetic variation in a Mediterranean germplasm collection, including Italian cultivars largely cultivated in Apulia, three sets of cultivars derived from Algeria, Syria and Tunisia, and a panel of autochthonous Apulian genotypes collected in the area of Salento. Some Apulian accessions were directly provided by farmers and could not be associated with known cultivars therefore they were referred to as unknown. The main aim of our study was to assess genetic structures and genetic relationships within the collection, and determine the origin and ancestry of Apulian autochthonous germplasm in relation to cultivars widespread in Italy and at the international level.

To characterize the panel of 218 genotypes, this study used nine SSR markers, widely used to describe olive genetic variation [14,28] (Supplementary Table S4). The PIC values associated with the markers indicate that microsatellites are highly polymorphic and informative to discriminate genotypes from Italy and Salento, in accordance with the results of previous studies [14,18,45]. The average heterozygosity of the collection was high, as previous observed by other authors [46,47]. However, it was lower than expected for a species like the olive, which is open-pollinated and subjected to somatic variation. This result could be explained by partial self-compatibility occurring in some olive cultivars, as reported by Montemurro et al. [48]. Moreover, the olive has been subjected to strong selective pressure [49], that can partially explain defects in heterozygosity. Prior to our work, other studies report lower heterozygosity than expected in Mediterranean olive germplasm [18,50,51]. Considering allelic diversity within olive populations originating from different geographical areas (Figure 2B), it is clear that Italian and Apulian germplasm display higher levels of variation, although our results might have been affected by a different number of genotypes sampled in different areas. The high level of variation in Italian germplasm is expected considering that, at the best of the authors’ knowledge, Italy is the country with the largest number of cultivars (600) compared to Algeria, Syria and Tunisia [12]. Algeria counts approximately 150 olive cultivars according to Hauville [52], while recent studies recognize the existence of only 36 varieties, with Chemlal and Sigoise being predominant throughout the country [28,53]. Similarly, genotypes cultivated in Syria and Tunisia might be less than reported due to several cases of sinonymy [54,55]. 

To analyse the genetic structure of our olive collection, this study chose to adopt three different methods, i.e. the model implemented by the software STRUCTURE, DAPC and hierarchical clustering. STRUCTURE is widely used to assess stratification and assign individuals to *a priori* defined populations in outbreeding species [11,26,27,35,36,39]. However, the method assumes that the population is panmictic, thus might not be ideal for a clonal or a partially clonal species such as the olive [56] and should be complemented by non-parametric methods such as DAPC and hierarchical clustering.

For K = 4, the STRUCTURE analysis assigned Algerian and widespread Italian cultivars to two separate subpopulations (SP1^a^ and SP1^b^, respectively), while the accessions collected in Apulia occurred in two further groups, SP2^a^ and SP2^b^, together with Tunisian and Syrian genotypes, respectively. At K = 7, the STRUCTURE subpopulations largely overlapped with clusters identified by DAPC analysis. In more detail, besides SP1^a^ and SP1^b^, the others were further separated according to geographical origin. In addition, the accessions of Apulia were divided into three different groups (SP2^a^’, SP2^b^’’ and SP2^b^’’’). This result was also explored in view of hierarchical clustering, which offers a more detailed view of diversity between accessions, showing different levels of relationships and disclosing also synonymies. Three main groups were observed. The national olive germplasm separates from the remaining cultivars, which were divided in two main clades: The first includes Algerian, Syrian and the half of Apulia accessions, while the second one the Tunisian and the other half genotypes collected in Apulia.

Our results indicate a high level of genetic variation of Apulian germplasm. Indeed, for K = 7, the STRUCTURE analysis assigned Apulian germplasm to three subpopulations. The subpopulation SP2^a’^ includes the majority of accessions related to Leccino, such as Leccino_Castellana, Leccino_Nuzzaci_LE, Leccino_Gervasi_LE, Leccino_LE and Avellona_LE, and four unknown accessions were shown to display reduced OQDS symptoms. SP2^a’^ also includes the cultivars Taggiasca, Cima di Bitonto, Ogliarola Salentina and the Syrian cultivar Dan, previously found similar to Italian genotypes [18]. The group SP2^a’^ is genetically far from the other sub-populations, except for SP2^b’’’^ (Figure 3), which includes, besides widely cultivated Italian cultivars such as Dritta, Simone, Nocella and Toscanina, also accessions collected in Salento (such as Cellina di Nardò and several unknown genotypes). Interestingly, SP2^b’’’^ also includes the two Fs-17 genotypes showing resistance to OQDS, and the accession Unknown_Toll2_Vernole_LE, asymptomatic to OQDS. These results suggest the possibility to find, in local germplasm, other genotypes tolerant to OQDS which can be adapted to the environmental conditions of Salento. Finally, a third group (SP2^b’’^) only includes genotypes collected in Salento (except for Termine di Bitetto and Cascata) and shows the lowest differentiation.

Overall, the genetic differentiation of the whole Apulian germplasm is lower than other populations (Figure 2B), indicating the composition of more genetic patterns. 

This study highlights that Italian olive germplasm can be associated with different genetic clusters, while Algerian, Syrian and Tunisian cultivars mostly refer to single gene pools (Figure 2B). In addition, genetic clusters identified in Italian genotypes are nearly absent in the other Mediterranean countries investigated in this work. This can be explained by the intensive gene flow that occurred in Italy, historically subjected to numerous events of colonization and human migration. In addition, Italian and Apulian populations show different genetic patterns, suggesting that germplasm with different origin might have been introduced in Italy in independent waves, and then might have gradually mixed. It is well known that at least two events of domestication occurred in the olive history, generating two notable gene pools, corresponding to Q3 in Levant and Q2 in central Mediterranean Basin, while a third gene pool was recognized in the western zone (Q1) [8,46]. It is possible that Apulian olive germplasm has been subjected to a great Greek influence, given the proximity to the Aegean Islands, and then, vegetative propagation, crossing, human migration and artificial selection generated the broad variability.

Surprisingly, the unknown genotypes collected in the area of Salento were shown to be in most cases genetically distinct from known cultivars, suggesting that they may be landraces selected by farmers on the basis of morpho-agronomic traits and adaptation to environmental conditions, or the result of hybridization among olive cultivars. This germplasm, in some cases, was shown to be asymptomatic to OQDS, and could represent a valuable source to investigate through new smart-breeding techniques [57], in order to discover genotypes and allelic variants of value in the current Apulian olive scenario. 

## 4. Materials and Methods

### 4.1. Plant Material

This study analyzed a panel of 218 olive accessions from Italy (149), Algeria (23), Syria (19) and Tunisia (27) (Supplementary Table S2). In more detail, 65 Italian cultivars (under code ITA) were collected in a pre-moltiplication field located in Palagiano (Taranto, Italy), in the framework of the REGEROP project. This panel includes cultivars which are widespread in Italy and Apulia. Eighty-four samples (under code LE) were collected in the area of Salento, including five genotypes with no assigned name. These genotypes represented asymptomatic exceptions in fields heavily affected by the OQDS (Figure 1) after monitoring and evaluating in accordance to the procedure reported in the EPPO Bulletin focused on diagnostics of X. *fastidiosa* (48/2018). These plants were assumed tolerant/resistant to the disease. Algerian germplasm (under code ALG) derived from the Institut Technique de l’Arboriculture Fruitière et de la Vigne (ITAFV, Takarietz, Bejaia, Algeria). The Syrian accessions (under code SYR) were sampled in 2005 in the region of Aleppo by the General Commission for Scientific Agricultural Research (GCSAR), centre of Aleppo. The Tunisian genotypes (under code TUN) were collected from the experimental fields located at the Olive Tree Institute of Sfax (OTI, Tunisia). 

### 4.2. SSR Molecular Analysis

The genomic DNA was extracted from 70 mg of lyophilized young leaves, according to the protocol reported by di Rienzo et al. [14]. The DNA quality and concentration were checked on 1% agarose gel. The panel of 218 olive accessions was genotyped by using nine informative nuclear SSR (Supplementary Table S4) [14,28]. The amplification products were detected by the automatic capillary sequencer ABI PRISM 3100 Avant Genetic Analyzer (Applied Biosystems, Foster City, CA, USA), and marker scoring was carried out using the GeneMapper genotyping software v3.7 (Thermofisher — Applied Biosys 2.1. Foster City, USA). The GeneScan TM 500 LIZ TM dye Size Standard (Applied Biosystem, USA) was used as internal molecular weight standard. 

### 4.3. Molecular Marker Diversity and Population Structure

The molecular marker diversity was investigated through different genetic indices, i.e. number of alleles (Na), observed (H_o_) and expected (H_e_) heterozygosity and fixation index (F) [58], which were calculated using the software GENALEX v.6.5 (http://anu.edu.au./BoZo/GenAIEx) [59]. The polymorphic index content (PIC) [60], indicating the informativeness of SSR primer combinations, was calculated using the software Cervus v.2.0 [61].

Pairwise relatedness was also used to calculate the allelic similarity for codominant data using GenAlEx 6.501, by using the Lynch and Ritland estimator (LRM) [62]. 

The population genetic structure analysis was performed using three approaches. The first was the Bayesian model-based clustering method implemented by the software STRUCTURE v.2.3.4 (http://pritch. 258 bsd.uchicago.edu/structure.html) [63]. To evaluate the number of olive sub-populations (K) best fitting with molecular data, for each K ranging from 1 to 10, ten independent runs were performed, using 100,000 MCMC repetitions and 100,000 burn-in periods. The resulting data were analysed by the Structure Harvester software [64], which is based on the *ad hoc* ∆K statistics [65]. The accessions were assigned to a specific subpopulation if the value of the corresponding membership coefficient (qi) was higher than 0.6, otherwise they were considered admixed. Based on the subpopulations defined by the STRUCTURE analysis, the F_ST_ index for pairwise comparisons was calculated using Genalex v.6.5.

The discriminant analysis of principal components (DAPC) was used as complementary clustering methods to analyze genetic structure. The DAPC is a multivariate method that uses a non-hierarchical approach for defining genetic clusters. The DAPC was implemented in the adegenet package for the R statistical environment [56].

Finally, the genetic structure and genetic relationships between individual cultivars were assessed using a weighted neighbor-joining method, using a dissimilarity matrix, through the software DARWIN v. 6.0.010 (http://darwin.cirad.fr), using bootstrapping with 1000 replicates to determine support for each node [66].

## Figures and Tables

**Table 1:** The diversity indices of 9 simple sequence repeat (SSR) markers detected in 218 olive accessions collected in Algeria, Tunisia, Syria and Italy.

Locus	Na	Ne	Ho	He	PIC	F
DCA03	15	7.70	0.88	0.87	0.86	−0.02
DCA05	12	6.46	0.78	0.85	0.83	0.08
EMOL	17	3.49	0.32	0.71	0.92	0.56
DCA18	32	10.90	0.75	0.91	0.72	0.17
DCA09	23	13.63	0.84	0.93	0.86	0.1
DCA15	20	3.64	0.42	0.73	0.9	0.43
GAPU101	22	8.44	0.83	0.88	0.87	0.06
DCA17	30	7.44	0.57	0.87	0.87	0.34
EMO90	18	8.09	0.78	0.88	0.69	0.11
Total	189	69.78				
Mean	21	7.75	0.68	0.85	0.83	0.2

## Supplementary Materials

The following are available online at https://www.mdpi.com/2223-7747/8/8/268/s1, Table S1: Genetics diversity indices of 9 SSR markers detected for each of the five populations based on geographical origin. Table S2: List of olive accessions collected in different areas of the Mediterranean basin and genotyped in this study with SSR markers. For each genotype, geographical origin and clustering groups were based on the STRUCTURE model and a discriminant analysis of principal components are reported. Table S3: List of olive synonymies detected on the basis of the LRM estimator. The cut-off threshold used was established at LRM = 0.35. Table S4: List of the 9 microsatellite markers (SSR) tested in this study. For each SSR, the identification code (SSR ID), bibliographic reference, repeat motif, primer sequence and annealing temperature are reported. Figure S1: (a). The mean of estimation ln probabilistic data of the Olive in the Mediterranean basin (b). A graph of delta K values to determine the best number of populations present in olive germplasm collection. The best K was at K = 7. Figure S2: Supporting data for DAPC analysis. (a) A comparison of clustering solutions by the Bayesian information criterion (b) A barplot of eigenvalues for linear discriminant functions. Figure S3: The genetic structure assessed by discriminant analysis of principal components (DAPC). The 3-D scatter plot is referred to the fourth, fifth and sixth discriminant functions. Figure S4: Frequency distribution of values of the Lynch and Ritland estimator (LRM) across olive germplasm.
