# Supplementary figures and images for: Genetic Characterization of Apulian Olive Germplasm as Potential Source in New Breeding Programs
Source: Plants (Basel). 2019 Aug 5;8(8):268. doi: 10.3390/plants8080268 (PMC6724140; doi:10.3390/plants8080268)

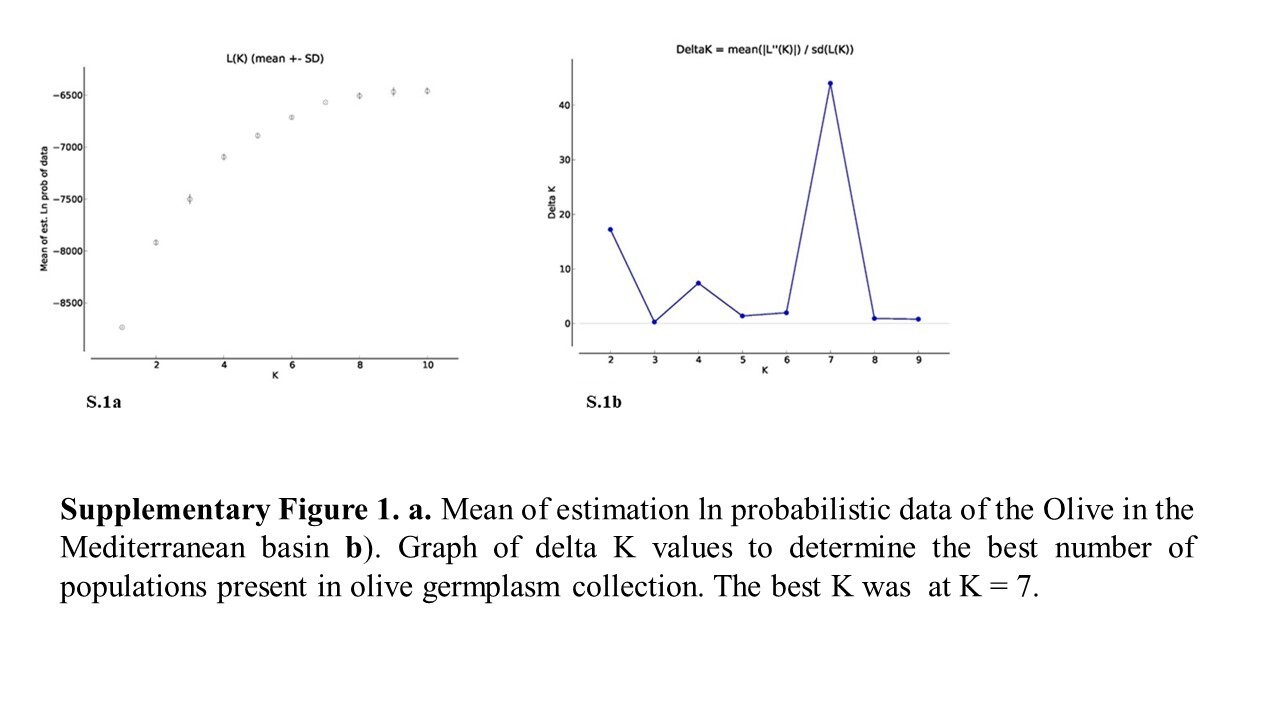

Supplement: Supplementary file 1 [file plants-08-00268-s001.zip › Supplementary-Figure_S1.jpg]

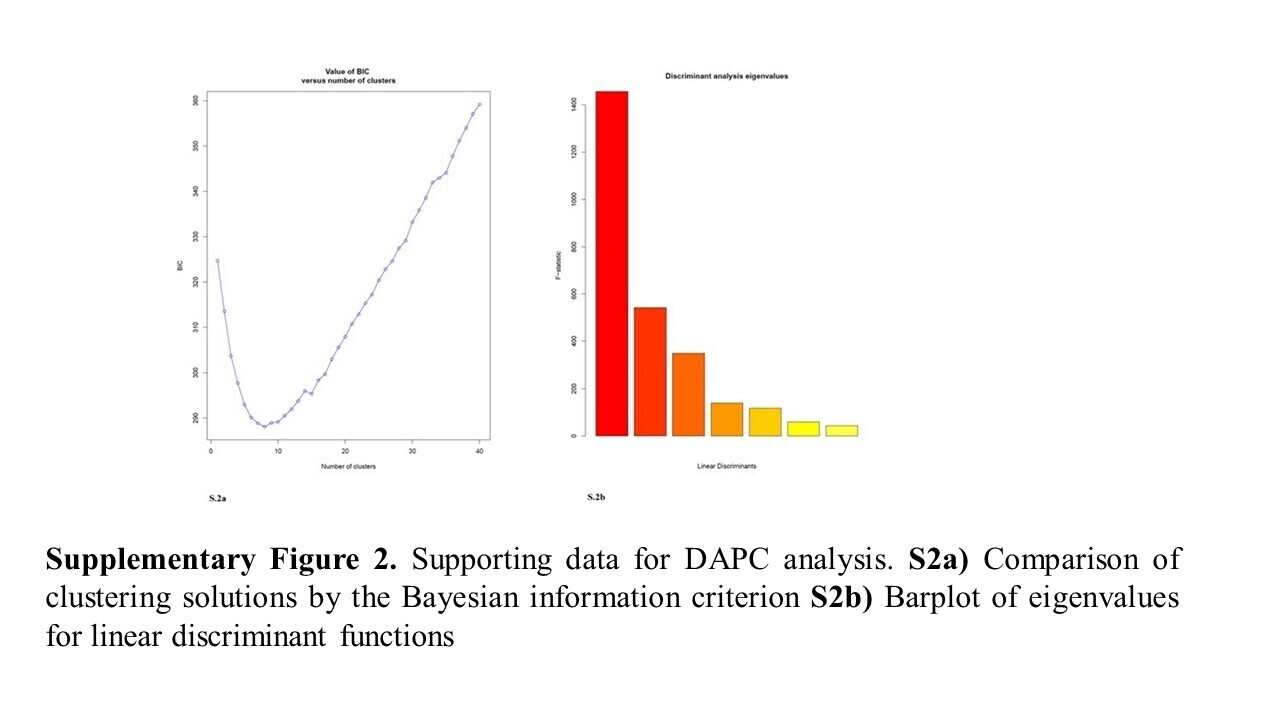

Supplement: Supplementary file 1 [file plants-08-00268-s001.zip › Supplementary-Figure_S2.jpg]

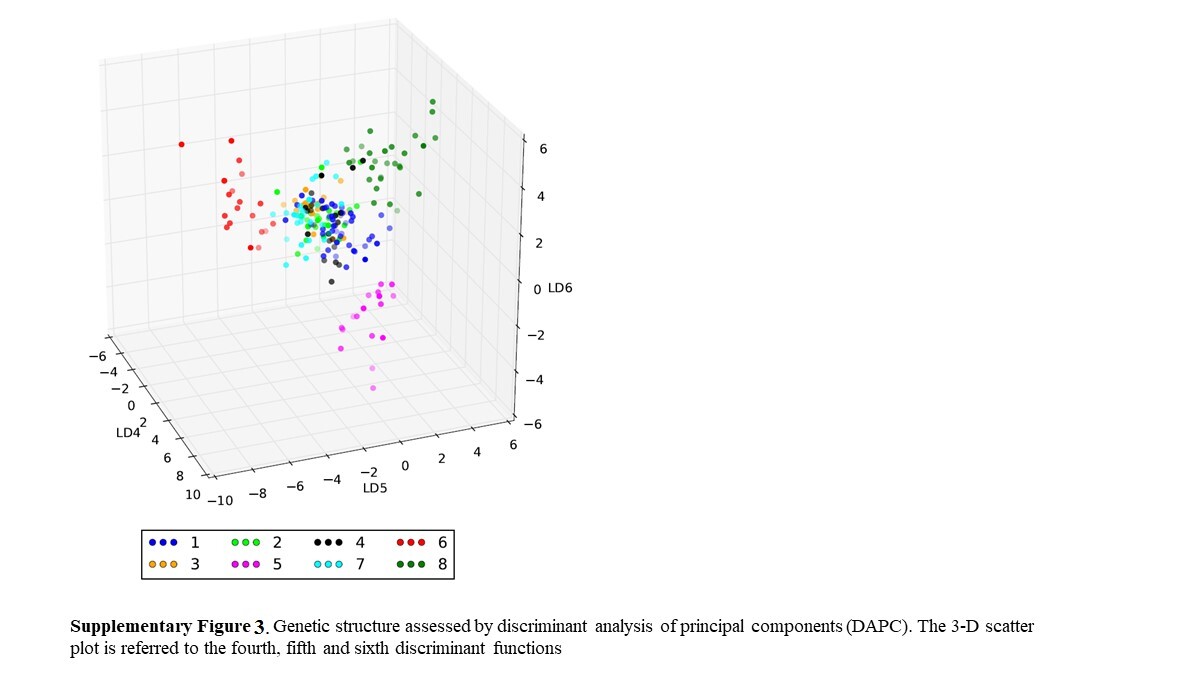

Supplement: Supplementary file 1 [file plants-08-00268-s001.zip › supplementary-Figure_S3.jpg]

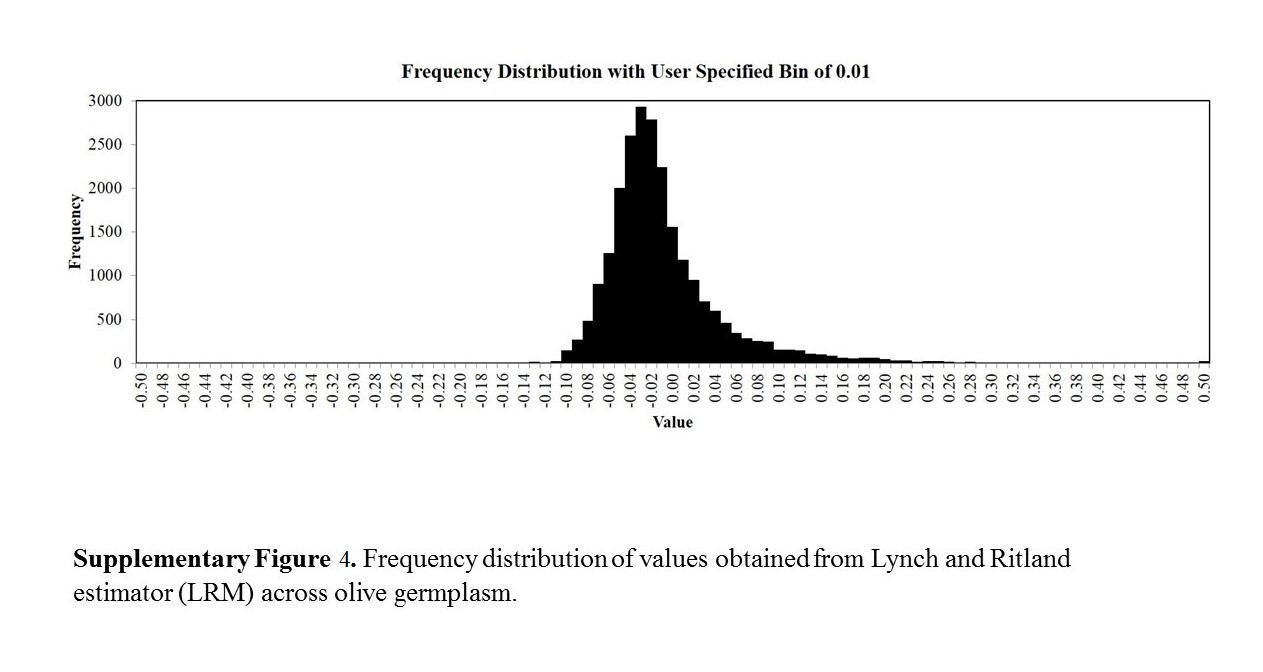

Supplement: Supplementary file 1 [file plants-08-00268-s001.zip › Supplementary-Figure_S4.jpg]
